# Phytochemical screening and antioxidant profiling of Sumatran wild mangoes ( *Mangifera* spp.): a potential source for medicine antidegenerative effects

**DOI:** 10.12688/f1000research.22380.3

**Published:** 2020-06-30

**Authors:** Fitmawati Fitmawati, Esi Resida, Sri Nur Kholifah, Rodesia Mustika Roza, Muhammad Almurdani, Emrizal Emrizal

**Affiliations:** 1 Department of Biology, Faculty of Mathematics and Natural Sciences, Universitas Riau, Pekanbaru, Riau, 28293, Indonesia; 2 Department of Chemistry, Faculty of Mathematics and Natural Sciences, Universitas Riau, Pekanbaru, Riau, 28293, Indonesia; 3 School of Riau Pharmacy, Pekanbaru, Riau, 28293, Indonesia

**Keywords:** antioxidant, gallic acid, quercetin, Sumatran, wild mango

## Abstract

**Background:** New findings on the potential of wild mangoes from the island of Sumatra as a source of antioxidant helps their conservation effort as it introduces their useful compounds to the public. This study aims to analyze the antioxidant profile and quantification of gallic acid and quercetin content from leaves and bark of Sumatran wild mangoes. Exploration and analysis of phytochemical constituents from 11 Sumatran wild mangoes was performed.

**Methods: **Antioxidant activity of wild mangoes was analysed with 1,1- diphenyl-2-picryl hydroxyl (DPPH), and determination of quercetin and gallic acid content was performed by high performance liquid chromatography (HPLC) method. Total flavonoid and phenolic analysis was also performed. Curve fitting analysis used a linear regression approach.

**Results:** The highest level of antioxidant activity, phenolic compound and flavonoid compound was found in the leaves and bark of *Mangifera *sp1. (MBS), the bark of *M. foetida*_3_ (var. batu) and leaves of *M. torquenda*, and the bark and leaves of *M. sumatrana*, respectively. The content of gallic acid in leaves ranged from 5.23-35.48 mg/g dry weight. Quercetin content of wild mangoes leaves ranged from 0.76 to 1.16 mg/g dry weight with the lowest value in *M. foetida*_2_ (var. manis) and the highest in *M. laurina*.

**Conclusion:** The results obtained are expected to be useful in supporting the development of drugs that have antidegenerative effects.

## Introduction

Sumatran wild mangoes have the potential to be used as an herbal medicine and are found in the Sumatra rainforests. However, they are threatened with extinction due to rapid and massive deforestation, and due to this species lower economic market value and low consumption by humans, there is little interest in their cultivation ^1^. This could lead to an increase in the number of endangered species in the Sumatran rainforest. In nature, these species are ecologically valuable as the primary food for primates and other wildlife.

There are eight wild mango species found in Sumatra: *Mangifera quadrifida*, *M. torquenda*, *M. magnifica*, *M. grifithii*, *M. kemanga*, *M. sumatrana*, *Mangifera* sp1. (MBS) and *Mangifera* sp2. (MH) ^2, 3^. The conservation of wild mango types will not be effective if the benefits of these plants are unknown, while their unique characteristics show that these plants have the potential to be therapeutic agents with high antioxidant ability ^4^. Consequently, studying their ecological role and finding their potential biomedicine compounds is the best way to aid wild mango conservation.

These wild mangoes are acidic and have a strong turpentine aroma, rough fibers and strong odor, showing that these mangos are an antioxidant source as reported by Fitwawati *et al.* ^4^. Grundhofer *et al.* ^5^ Explains that mango is a rich source of many phytochemical compounds. Main compounds such as phenolic and flavonoid can obtained from various parts such as fruit, kernel, leaves, and bark ^6, 7^. Mohan *et al.* ^8^ mentioned that mango leaves and bark contains phenolic were very high which were responsible for various pharmaceutical activities, as antioxidant. Antioxidants are vital to resolve some types of degenerative diseases, improving immunity and responding to external attacks, such as bacteria, viruses, fungi, and various disease-causing germs. They may even be used to manage chronic diseases, such as cancer. In the study by Taing *et al.* ^9^, skin and flesh extracts of mangos ( *M. indica*) had the remarkable action of inhibiting proliferation of breast and colon cancer cells ^10^.

Previous studies show that mango skin ( *M. indica*) possess flavonol O- and xanthone C-glycosides ^11^, gallotannins and benzophenone derivatives ^12^. Mango seeds contained bioactive components such as phenolics, carotenoids and ascorbic acid ^13^, carbohydrates (58–80%), proteins (6–13%), essential amino acids and lipids (6–16%) ^14^. The polyphenol component of mango extract is phenolic acid (gallic acid) and flavonoids (quercetin) ^15^. These compounds are found in the edible fruits and have been shown to have great potential for protecting the body from oxidative stress-associated damages ^16, 17^.

Gallic acid (3,4,5-trihydroxybenzoid acid) is a major polyphenolic compound present in mangoes ^18^. Phenolic compounds are vital in the immune system response to chronic degenerative diseases, such as anti-aging, anti-inflammatory, antidiabetic and antiproliferative activities, and have a cardioprotective effect, including reduction the side effects of chemotherapeutics ^19^. Quercetin (3,3’4’,5,7-pentahydroxyflavone) is one of the most abundant flavonoid-derived food compounds and is present in various mangos (e.g. *M. indica*) ^20^. Flavonoid structure contains a double bond in the C ring and a 4-oxo group, which enhances its potent antioxidant activity ^21^. Flavonoids have been scientifically proven to be anti-allergic, anti-inflammatory, anti-cancer ^22^, antimicrobial ^23^ and antiviral ^24^. Quercetin generally exists in edible plants and is mostly used in the production of traditional medicine to relieve some type of diseases ^25– 27^.

Previous research related to the antioxidant potential of wild mango species from Sumatra had been carried out qualitatively by Fitmawati *et al.* ^4^. Yet, this research topic needs the support of quantitative data and detailed metabolite content in order to clarify the antioxidant compound of wild mangos that have high potential to be a source of antidegenerative drugs. To the best of our knowledge, quantitative analysis of the antioxidants in wild mangos has not yet been reported. Therefore, this study aimed to analyze the antioxidant compound (gallic acid and quercetin) of wild mangos from Sumatra, Indonesia in order to preserve its existence in nature. The results obtained are expected to be advantageous in the effort of discovering types that contain highly phenolic and flavonoid compounds, which will support antidegenerative drug development for public health. The finding of medicinal compounds in wild mangos may make stakeholders pay attention to cultivation and restoration, so their population in nature will be conserved.

## Methods

### Equipment and materials

***Equipment.*** Preparative-plate glass for thin layer chromatography, *Whatman* filter-paper, vacuum rotary evaporator, ultraviolet lamps 254 and 366, UV-Visible spectrophotometer (GENESYS 10S UV-VIS), High Performance Liquid Chromatography (HPLC; Shimadzu LC 20AD), cuvette, micropipette. *Materials*: stem bark and leaves of wild mango from Sumatra, distilled water (Batraco), ethanol (Batraco; as solvent), silica gel GF254 (Batraco; for isolation), and ethyl-acetate (Batraco; as solvent).

### Plant preparation and extraction of wild mangoes

**Figure 1.**
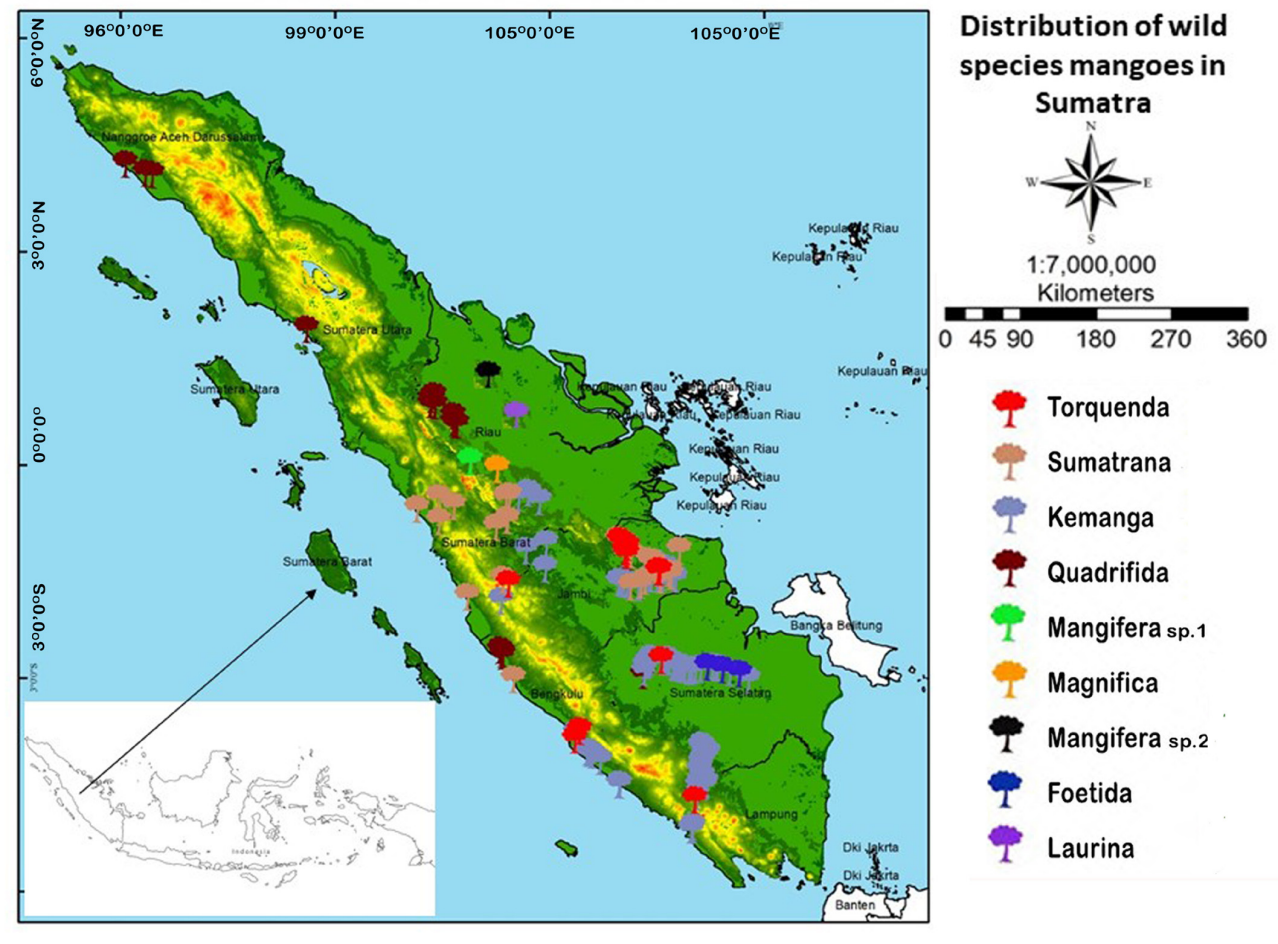
Distribution of the wild mango sources involved in this study.

The wild mango samples were collected from several provinces in Sumatra Island, such as Riau, Jambi and South Sumatera, as depicted in Figure 1. Wild mango bark was dried in an oven at 40°C, while the leaves were freeze-dried. Dried bark and leaves were ground by a blender and ~100 g of the resulting powder was macerated with 200 ml ethanol until it was submerged, and was then soaked for a day. All macerate was collected and evaporated with a rotary vacuum evaporator at 50°C to obtain a solid-liquid extract.

### Quantification of antioxidant content

Antioxidant activity analysis was assessed with the DPPH(2,2-diphenyl-1-picryl-hydrazyl-hydrate) method ^28^. Briefly, approximately 2 mg of the dried sample was dissolved in 2 mL of methanol until the concentration reached 1000 mg/mL. Then, in a plate consisting of rows A-H (each row consisted of 12 wells), 100 mL of sample was added to each well in row A. Then, a total of 50 mL methanol was added to each well for rows B to F. 50 mL was taken from row A and then added to the row B; the same volume taken from row B and added to row C; the same procedure was done to row F. 50 mL was taken from row F and discarded. The following concentrations were obtained through this process: 1000 (row A), 500 (B), 250 (C), 125 (D), 62.5 (E), and 31.25 (F) g/mL. Row G to H were filled with 50 mL of methanol, only wells 1-6 of row H were filled. Furthermore, for DPPH test, additional methanol of 80 mL (concentration of 80 mg/mL) is added to the rows A to G, and then incubated for 30 minutes. Radical bounding was measured by the decrease in DPPH absorbance using a microplate reader at a wavelength of 517 nm. A positive control was used as a comparison (ascorbic acid, 50 ug/mL).

The antioxidant activity is presented as inhibition percentage of IC _50_, which was calculated by the formula below ^29^:

$$\%\text{Inhibition}=\left(\frac{A_{0}-A_{i}}{A_{0}}\right)\times 100\%\;(1)$$

where *A _0_* is the absorbance without sample and *A _i_* is the absorbance of the tested sample.

### Quantification of total flavonoid content

Analysis of total flavonoid content was carried out using the method of Xu and Chang ^30^. Quercetin solution was firstly dissolved into five concentrations (20, 40, 60, 80 and 100µg/ml) for a standard curve. Subsequently, ~100 μl of quercetin (blank) sample was mixed together with 50 NaNO _2_ 5% and 50 μl AlCl _3_.6H _2_O (aluminum chloride hexahydrate) 10% and 50 μl NaOH 1 M were added to a 96 well clear polystyrene microplate.The mixture was incubated in a dark place at room temperature for 30 minutes. After that, the mixture was assessed using a microplate reader at a wavelength of 510 nm. Total flavonoid content is determined by following equation:

$$M=\frac{bD_{p}V}{m_{t}}\left(\frac{m_{total}}{m_{dry}}\right)\;(2)$$

where *b* and *D _p_* are regression coefficient and dilution factor, respectively, *V* is extracted volume of the sample, and *m _t_* is extracted mass. Note that the mango sample is in dried form and mass of 100 gr, so that *m _total_* refers to total mass of extracted sample and m _dry_ indicates sample dry mass.

### Quantification of total phenolic content

Analysis of total phenolic contents from dried samples of 100 gr was performed using the method of Folin-Ciocalteu from Musa *et al.* ^31^. Gallic acid solution was initially dissolved into five concentrations (20, 40, 60, 80, and 100µg/ml) for the standard curve. About 100 μl of hydrolysis and non-hydrolysis of gallic acid as reference sample was then mixed with 50 μl of Folin-Ciocalteu reagent 0.25 in a 96 well clear polystyrene microplate. Next, 50 μl Na _2_CO _3_ 7,5% was added to each well. The mixture was subsequently incubated in a dark place at room temperature for 30 minutes and was read using a microplate reader at a wavelength of 765 nm. Total content of phenolic is calculated by the same equation used for determining flavonoid content as in Equation 2.

### Quantification of quercetin and gallic acid content by HPLC

Mango extract was dissolved in ethanol (100 g/100 mL). Quercetin and gallic acid standards purchased from Sigma-Aldrich.These standard compounds were each prepared in several concentrations of 100, 50, 25, and 12.5 ug/ml. Each standard compound was mixed with 20 μL and eluted for 20 minutes through elution gradient method (with water/methanol = 0-100 and UV detector with wavelengths of 360 and 370 nm) to obtain a standard chromatogram. Chromatographic analysis was performed using an analytical scale (4.6 x 150mm) Shimadzu ODS C18 HPLC column with a particle size of 0.5 μm (flow rate 0.75 mL/min). The sample was then filtered using a 0.45 μm filter (13mm PTFE) and analyzed according to the HPLC method of quercetin and gallic acid. The level of quercetin and gallic acid contained in each sample was calculated from regression plot of Y= a lnX+b. This plot is described the ratio of the sample concentration to the standard chromatogram of the quercetin and gallic acid standard.

### Data analysis

The content of gallic acid and quercetin in the sample is indicated by the change of the solution color in DPPH test which indicated antioxidant activity ^32^. This change was read by a microplate (Berthold, Germany) using the software of MikroWin 2000 version 4.3x. Furthermore, a linear regression curve is performed to obtained quantification of gallic acid and quercetin content from each sample.

## Results

### Antioxidant content

Antioxidant values in this study were determined using the DPPH method. This method is a simple, easy, fast and sensitive method. Only a small amount of natural material is used to evaluate antioxidant activity, so it is widely used to test the ability of compounds that act as electron donors ^32^. The principle of this measurement is the presence of stable free radicals, namely DPPH mixed with antioxidant compounds that have the ability to donate hydrogen so that free radicals are neutralized ^33^. Measurement of antioxidant activity using UV-vis spectrophotometry is used so that the value of free radical will be known, expressed by the value of IC _50_ (inhibitory concentration). Table 1 contains the antioxidant activity of eight wild mango species.

**Table 1.** IC _50_ of wild mango species from Sumatran, Indonesia, using the DPPH (1,1- diphenyl-2-picryl hydrazyl) method.

Species		IC _50_ value (ppm)
*Mangifera foetida* (var. limus)	Bark	60.52
	Leaves	196.24
*Mangifera foetida* (var. manis)	Bark	117.62
	Leaves	195.12
*Mangifera foetida* (var. batu)	Bark	34.22
	Leaves	119.66
*Mangifera kemanga*	Bark	7.034
	Leaves	152.23
*Mangifera sumatrana*	Bark	10.57
	Leaves	8.70
*Mangifera laurina*	Bark	22.51
	Leaves	18.25
*Mangifera* sp1. (MBS)	Bark	33.24
	Leaves	0.88
*Mangifera* sp2. (MH)	Bark	45.48
	Leaves	2.40
*Mangifera quadrifida*	Bark	28.44
	Leaves	26.26
*Mangifera torquenda*	Bark	66.87
	Leaves	47.62
*Mangifera magnifica*	Bark	41.75
	Leaves	6.76

Antioxidant compounds will generally react to DPPH radicals through mechanism of donation of hydrogen atoms which causes the discoloration from purple to yellow ^32^. In this study, the strongest levels of antioxidant activity in wild mangos was found in *Mangifera* sp1. (MBS) leaves and bark extract with IC _50_ value of 0.88 ppm and 33.24 ppm, respectively. Antioxidant compounds at a moderate level were discovered in the extract of *Mangifera foetida*_1_ (var. limus) leaves (IC _50_ value of 196.24 ppm) and *Mangifera foetida _2_* (var. manis) bark (IC _50_ value of 117.62 ppm).

### Phenolic and flavonoid content

The phenolic and flavonoid compounds were quantified in the mangos using the method of Folin-Ciocalteu from Musa *et al.* ^31^ and the method of Xu and Chang ^30^, respectively. Total phenolic content was represented in milligrams (mg) of gallic acid, which was equivalent to per gram of the dry weight of mango leaves (mg GAE/g) ( Table 2). Total flavonoid content was represented in milligrams (mg) of quercetin, which was equivalent to per gram of the dry weight of mango leaves dry weight (mg of QE/g).

**Table 2.** Total phenolic and flavonoid content of wild mango species from Sumatran, Indonesia.

Species		Total phenolic content (mg GAE/g)	Total flavonoid content (mg QE/g)
*M. foetida _1_* (var. limus)	Bark	41.76 ± 0.29	63.93±0.39
	Leaves	24.64±0.14	57.29±0.26
*M. foetida _2_* (var. manis)	Bark	75.36 ± 0.10	68.93±0.16
	Leaves	63.59±0.18	63.82±0.24
*M. foetida _3_* (var. batu)	Bark	100.65 ± 1.94	94.34±0.24
	Leaves	70.83±0.36	92.50±0.41
*M. kemanga*	Bark	90.65 ± 0.59	87.46±0.35
	Leaves	20.08±0.18	48.55±1.55
*M. Sumatrana*	Bark	88.43 ± 1.82	98.69±0.89
	Leaves	65.72±0.23	107.50±3.93
*M. laurina*	Bark	44.10 ± 2.97	39.00±0.21
	Leaves	81.07±1.40	95.36±0.41
*M.*sp _1_. (MBS)	Bark	84.80 ± 6.60	68.30±0.44
	Leaves	69.63±0.27	81.55±3.92
*M.* sp _2_. (MH)	Bark	93.01 ± 0.41	82.92±0.06
	Leaves	64.70±0.19	96.72±1.36
*M. quadrifida*	Bark	79.72 ±0.18	58.85±0.24
	Leaves	89.00±0.77	75.53±0.27
*M. torquenda*	Bark	76.30±0.18	85.10±0.21
	Leaves	92.48±0.14	66.65±0.35
*M. magnifica*	Bark	77,37±0.50	66.55±0.14
	Leaves	45.63±0.44	65.70±3.71

### Gallic acid and quercetin content

**Figure 2.**
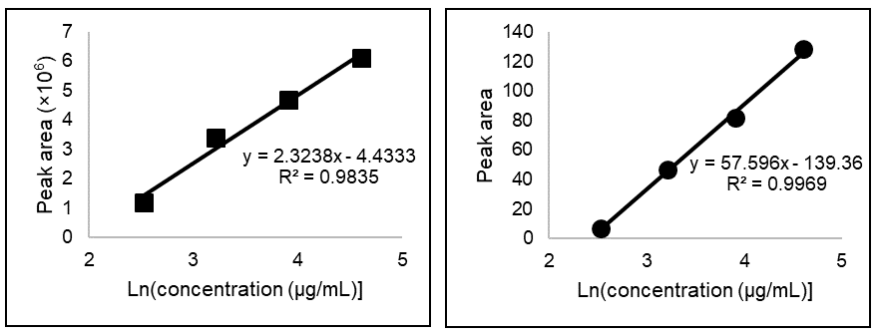
Plot of standard calibration of quercetin (left) and gallic acid (right).

Spectrum chromatogram of standard solutions and chromatograms of each species at wavelengths of 360 and 370 nm were used. Specific quantitative tests of gallic acid and quercetin on the leaves of wild mangos from Sumatra using HPLC showed that the plant leaves were positive for both bioactive compounds. The plot of standard calibration fo quercetin and gallic acid is shown in Figure 2 and the results are summarized in Table 3.

**Table 3.** Quercetin and gallic acid content of wild mango leaves from Sumatran, Indonesia.

Species	Peak area (a.u.)		Retention time (min)		Content (mg/g dry weight)	
	Quercetin	Gallic acid	Quercetin	Gallic acid	Quercetin	Gallic acid
*M. foetida _1_* (var. limus)	51153	144789	14.97	10.24	0.76	13.89
*M. foetida _2_* (var. manis)	138513	198816	15.64	10.49	0.79	35.48
*M. foetida _3_* (var. batu)	137886	123830	15.71	10.26	0.79	9.65
*M. kemanga*	550164	98147	15.47	10.26	0.97	6.18
*M. sumatrana*	714087	88518	15.45	10.26	1.06	5.23
*M. laurina*	902079	122354	15.43	10.23	1.16	9.41
*Mangifera* sp _1_. (MBS)	96833	115273	15.56	10.23	0.78	8.32
*Mangifera* sp _2_. (MH)	227010	128227	15.51	10.23	0.83	10.42
*M. quadrifida*	230653	113892	15.49	10.26	0.83	8.12
*M. torquenda*	462008	114851	15.50	10.28	0.93	8.26
*M. magnifica*	243010	111497	15.48	10.26	0.83	7.79

## Discussion

Antioxidants are a group of chemical compounds that have the function of suppressing cell damage, caused by free radicals, by giving electrons speedily and transforming the free radicals into stable forms, in order to prevent oxidative damage that cause ailments ^34^. The presence of antioxidants in plants is primarily as a protective compound from pest and disease, known as bioactive compounds. In this study, the quantitative phytochemical analysis was purposely conducted to determine the level of antioxidants and content of flavonoid and phenolic compounds, specifically gallic acid and quercetin. These analyses were performed using the DPPH method, and concentration of gallic acid and quercetin were analyzed using HPLC.

Based on the results of total antioxidant values of wild mangoes using DPPH assay, it was revealed that *Mangifera* sp1. (MBS) is the highest antioxidant activity amongst other wild mangos (leaves, 0.88 ppm; bark, 33.24 ppm). According to Badarinath ^35^, antioxidants are expected to be very strong if the IC _50_ value is smaller than 50, in the range of 50–100 ppm, yet it is weak when the IC _50_ value ranges between 100–250 ppm and is inactive if the IC _50_ is <250 ppm. The smaller the IC _50_ value, the higher the antioxidant activity. The antioxidants contained in mangos are essential for enhancing the immune system in the body, and are an immunomodulator agent ^36^, including analgesic ^37^, antimicrobial and antidiabetic properties ^38^. Fitmawati *et al.* ^39^ reported that powder ethanol extract of *Mangifera* sp1. (MBS) leaves are immunomodulatory agents with phagocytic activity at 69.67%. Ascorbic acid contained in the bark and leaves of mangos has great potential to heal chronic wounds and inflammation ^40^. Also, mangos are a particularly rich source of polyphenols, a diverse group of organic micronutrients found in plants, which exert specific health benefits.

The presence of gallic acid and quercetin from wild mangoes in this study shows that they have potential as antioxidants of phenolic and flavonoid groups. Generally the higher levels of phenolic and flavonoid compounds, the antioxidants values is also higher. Based on the results of this study antioxidants values is determined by total phenolic and flavonoid compounds, and not only their that contributing in antioxidants values. Research by Ou *et al.* ^41^ did not find a linear relationship between total phenolic with antioxidants values. Antioxidants values are not limited to phenolic or flavonoid compounds, so there is no simple relationship between total phenolic and flavonoid when comparing antioxidants values between plant extracts ^42^. The highest phenolic compounds of wild mangos were in the bark of *M. foetida* (var. batu) and leaves of *M. torquenda* (100.65 mg/g and 92.48 mg/g, respectively). Whereas, the lowest phenolic compounds of wild mangos were in the bark of *M. foetida* (var. limus) and the leaves of *M. kemanga* (41.76 mg/g and 20.08 mg/g).

In a previous study on the bark,old leaves, and young leaves of Van Dyke cultivated mango in Brazil, gallic acid content was 0.24 g/kg, 0.43 g/kg, and 3.49 g/kg, respectively ^15^. The results of the present study show that wild mangos have higher phenolic contents than cultivated mangoes, such as the Van Dyke mangos. With the abundant phenolic content, wild mangos from Sumatra have a potential source of natural antioxidant agents. Currently, herbal tea derived from mango leaves ( *M. indica*) has been developed and scientifically proved to be a source of mangiferin and other phenolic contents ^43, 44^. Therefore, phenolic contents found in wild mangos in this study may be expected to be further developed into healthcare products in the future.

The highest amount of flavonoid compounds were found in the bark and leaves of *M. sumatrana* (98.69 mg/g and 107.50 mg/g, respectively), and the lowest values were found in the bark of *M. laurina* and leaves of *M. kemanga* (39.00 mg/g and 48.55 mg/g, respectively). Barreto *et al.* ^15^ reported that quercetin pentoside compounds in methanol extract of leaves of Van Dyke cultivated mangos from Brazil was 1.33 g/kg in old leaves and 4.93 g/kg in young leaves. In contrast, pentoside was undetectable in the bark ^15^. However, the present study revealed that all bark of wild mangos assessed contained quercetin. Ali *et al.* ^45^ and Kim *et al.* ^18^ also reported that cultivated mangoes ( *M. indica*) mostly contain flavonoids, carotenoids, vitamin E and C, terpenoids and steroids.

Phenolic and flavonoid have many derivates. Mangiferin was a phenolic compound that very high levels in mango leaves and bark ^8^. Wauthoz *et al.* ^46^ reported that mango bark were contains protocatechic acid, catechin, mangiferin, alanine, glycine, γ-amino-butyric acid, kinic acid, shikimic acid, and the tetra cyclic triterpenoids cycloart-24-en-3p,26-diol,3-keto dammar-24(ES-en-2Os,26-diol,C-24) epimers of cycloart-25 en 3β,24,27-triol and cycloartan-3β, 24,27-triol. The main flavonoids present in mango were quercetin and catechin ^47^. The presence of other phenolic and flavonoid compounds that affect the amount of gallic acid and quercetin in mango leaves and bark.

Based on the results of the HPLC chromatogram, the contents of the bioactive compounds of wild mangos showed that the content of gallic acid obtained from wild mangoes leaves ranged from 5.23-35.48 mg/g of dry weight, where the lowest content was in *M. sumatrana* and the highest was *M. foetida* (var. manis). According to Soong and Barlow ^48^, mango seed extract ( *Mangifera indica* L.) contains gallic acid (23-838 mg/100 g of dry weight, equivalent to 0.23-8.38 mg/g). Rastraelli *et al.* ^49^ also stated that gallic acid content in mango bark is ~226.2 mg/100g of dry weight, 6.0 mg/100 g in seed kernel ^50^, and 6.9 mg/kg in mango pulp ^51^. These results indicate that gallic acid content is higher in wild mango leaves (as seen in the present study) compared to other parts of the mango.

The study by Barreto *et al.* ^15^ reported that gallic acid is the major secondary metabolite component in the flesh and seeds of mangoes ( *M. indica*). Gallic acid is the most abundant molecule in ‘Ataulfo’ mango peel ^52^. The flesh, seeds, and kernel of *M. indica* are sources of gallic acid, which is a bioactive compound with potential health-promoting activity ^53^.

Gallic acid has anticancer, anti-inflammatory, antimicrobial, and antimutagenic, including radical-scavenging, activities ^54^. It has also been shown to inhibit colon cancer cell (HCT-15) proliferation, inhibits platelet aggregation, calcium mobilization, and tyrosine protein phosphorylation in platelets ^20^, and inhibits inflammatory allergic reactions ^55^.

Quercetin plays a role in triggering fruit color development. Ajila and Prasada ^56^ stated that quercetin is the major flavonoid type in the ripe peel of mango. In the present study, *M. laurina* had the highest quercetin content, while the lowest was found in *M. foetida _1_* (var. limus). Quercetin content of wild mangoes leaves ranged from 0.76 to 1.16 mg/g of dry weight. Berardini *et al.* ^11^ reported that quercetin content in mango peel was 65.3 mg/kg of dry matter, equivalent to 0.653 mg/g dry weight. Based on these data, wild mangos leaves have higher quercetin content (as found in the present study) than peel. High quercetin content is found in fruits and quercetin-3-glucoside is mainly found in leaves ^20^.

Quercetin has various medicinal benefits, including aiding treatment in brain disorders, renal injury, cardiovascular diseases, high blood pressure, cancer, bacterial activity, inflammation, diabetes mellitus, arthritis and asthma ^57– 59^. It decreases estrogen receptor cells of breast cancer, inhibits tyrosine kinase, arrestshuman leukemic T cell development ^60^, protects the liver from oxidative damages ^61^, prevents cardiovascular disease, and exhibits antihistamine and anti-inflammatory effects associated with various forms of arthritis ^20^.

Fitmawati *et al.* ^39^ demonstrated that wild mangos leaves from Sumatra have immunostimulant activity and antioxidant compounds ^62^, including therapeutic efficacy potential for the prevention of degenerative diseases. Wild mangos from Sumatra contain flavonoids in high amounts, hence these contents need to be further explored and maximized for medicinal usage, in order to support the availability of medicinal resources. The results of the antioxidant activity using the DPPH method as assessed in the present study, and the total content of flavonoids and phenolics exhibited the varied results for each species. Wild mango species that have the highest antioxidant content may not have the highest total levels of flavonoids and phenolics because each plant species has different levels and types of metabolites.

## Conclusions

This study provided quantitative information about the presence of antioxidants, as well as the content of one of the main compunds from the flavonoid (quercetin) and phenolic (gallic acid) of wild mangos from Sumatra. The current investigation was undertaken to quantify the percentage of phytochemicals in the leaves and bark of wild mangos as an alternative raw material for medicine. The results obtained are expected to be useful in supporting the development of drugs that have anti-degenerative effects, as well as to support the conservation of wild mangos, which are rare while maintaining and improving their quality and diversity.

## Data availability

### Underlying data

Open Science Framework: Wild Mango Exploration in Sumatra for Potential Health Medicine, https://doi.org/10.17605/OSF.IO/MP6KF ^63^.

This project contains the following underlying data:

- Raw_Data_for_Gallic_Acid
- Raw_Data_for_Quercetin

Data are available under the terms of the Creative Commons Attribution 4.0 International license (CC-BY 4.0).

## References

[1] Fitmawati Harahap SP Sofiyanti N: Phylogenetic analysis of mango ( *Mangifera*) in Northern Sumatra based on gene sequences of cpDNA *trnL-F* intergenic spacer. *Biodiversitas.* 2017;18(2):715–719. 10.13057/biodiv/d180239

[2] Fitmawati Swita A Sofiyanti N: Keanekaragaman Mangga Sumatera Tengah. *Floribunda.* 2013;4(7):169–174.

[3] Fitmawati Zulkifli A Sofiyanti N: Spatial Distribution of Mango ( *Mangifera*) in East Sumatra Based on Land Cover and Altitude.Proceeding in International Conference on Plant Diversity (ICPD 2015) UNSOED, Purwokerto,2015 Reference Source

[4] Fitmawati Roza RM Isda MN: Kajian Potensi Terapeutik Mangga Liar ( *Mangifera.*spp.) Asal Riau Sebagai Agen Immunomodulator dan Profil Metabolomiknya.Research Progress Report on Scientific Research Grant, University of Riau,2018.

[5] Grundhofer P Niemetz R Schilling G: Biosynthesis and subcellular distribution of hydrolyzable tannins. *Phytochemistry.* 2001;57(6):915–927. 10.1016/s0031-9422(01)00099-1 11423141

[6] Ajila CM Naidu KA Bhat SG: Bioactive compounds and antioxidant potential of mango peel extract. *Food Chem.* 2007;105(3):982–988. 10.1016/j.foodchem.2007.04.052

[7] Dorta E Lobo MG Gonzalez M: Reutilization of mango byproducts: study of the effect of extraction solvent and temperature on their antioxidant properties. *J Food Sci.* 2012;77(1):C80–88. 10.1111/j.1750-3841.2011.02477.x 22132766

[8] Mohan CG Deepak M Viswanatha GL: Anti-oxidant and anti-inflammatory activity of leaf extracts and fractions of *Mangifera indica*. *Asian Pac J Trop Med.* 2013;6(4):311–314. 10.1016/S1995-7645(13)60062-0 23608334

[9] Taing MW Pierson JT Shaw PN: Mango fruit extracts differentially affect proliferation and intracellular calcium signalling in MCF-7 human breast cancer cells. *J Chem.* 2015;1–10. 10.1155/2015/613268

[10] Zhang H Zhang M Yu L: Antitumor activities of quercetin and quercetin-5',8-disulfonate in human colon and breast cancer cell lines. *Food Chem Toxicol.* 2012;50(5):1589–1599. 10.1016/j.fct.2012.01.025 22310237

[11] Berardini N Knodler M Schieber A: Utilization of mango peels as a source of pectin and polyphenolics. *Innov Food Sci Emerg Technol.* 2005;6:442–452. 10.1016/j.ifset.2005.06.004

[12] Berardini N Carle R Schieber A: Characterization of gallotannins and benzophenone derivatives from mango ( *Mangifera indica.* L. cv. Tommy Atkins) peels, pulp and kernels by high-performance liquid chromatography/electrospray ionization mass spectrometry. *Rapid Commun Mass Spectrom.* 2004;18(19):2208–2216. 10.1002/rcm.1611 15384138

[13] Jahurul MH Zaidul IS Ghafoor K: Mango ( *Mangifera indica* L.) by-products and their valuable components: a review. *Food Chem.* 2015;183:173–180. 10.1016/j.foodchem.2015.03.046 25863626

[14] Diarra SS: Potential of Mango ( *Mangifera indica.* L.) seed kernel as a feed ingredient for poultry: a review. *Worlds Poult Sci J.* 2014;70:279–288. 10.1017/S0043933914000294

[15] Barreto JC Trevisan MT Hull WE: Characterization and quantitation of polyphenolic compounds in bark, kernel, leaves, and peel of mango ( *Mangifera indica.* L.). *J Agric Food Chem.* 2008;56(14):5599–5610. 10.1021/jf800738r 18558692

[16] Materska M Perucka I Stochmal A: Quantitative and qualitative determination of flavonoids and phenolic acid derivatives from pericarp of hot pepper fruit cv. Bronowicka Ostra. *Pol J Food Nutr Sci.* 2008;12(2):72–76. Reference Source

[17] GutiErrez-Grijalva EP Ambriz-Pere DL Leyva-Lopez N: Review: dietary phenolic compounds, health benefits and bioaccessibility. *Arch Latinoam Nutr.* 2016;66(2):87–100. 29737665

[18] Kim H Moon JY Kim H: Antioxidant and antiproliferative activities of mango ( *Mangifera indica.* L.) flesh and peel. *Food Chem.* 2010;121(2):429–436. 10.1016/j.foodchem.2009.12.060

[19] Warpe VS Mali VR Arulmozhi S: Cardioprotective effect of ellagic acid on doxorubicin induced cardiotoxicity in wistar rats. *J Acute Med.* 2015;5(1):1–8. 10.1016/j.jacme.2015.02.003

[20] Masibo M He Q: Major mango polyphenols and their potential significance to human health. *Compr Rev Sci Food Saf.* 2008;7(7):309–319. 10.1111/j.1541-4337.2008.00047.x 33467788

[21] Torres-Leon C Rojas R Contreras-Esquivel JC: Mango seed: Functional and Nutritional Properties. *Trends Food Sci Technol.* 2016;55:109–117. 10.1016/j.tifs.2016.06.009

[22] Cazarolli LH Zanatta L Alberton EH: Flavonoids: prospective drug candidates. *Mini Rev Med Chem.* 2008;8(13):1429–1440. 10.2174/138955708786369564 18991758

[23] Manner S Skogman M Goeres D: Systematic exploration of natural and synthetic flavonoids for the inhibition of *Staphylococcus aureus* biofilms. *Int J Mol Sci.* 2013;14(10):19434–19451. 10.3390/ijms141019434 24071942 PMC3821565

[24] Cushnie TP Lamb AJ: Recent advances in understanding the antibacterial properties of flavonoids. *Int J Antimicrob Agents.* 2011;38(2):99–107. 10.1016/j.ijantimicag.2011.02.014 21514796

[25] Rivera L Morón R Sánchez M: Quercetin ameliorates metabolic syndrome and improves the inflammatory status in obese Zucker rats. *Obesity (Silver Spring).* 2008;16(9):2081–2087. 10.1038/oby.2008.315 18551111

[26] Torres PM Ortiz AR Villalobos MR: A comparative study of flavonoid analogues on streptozotocin-nicotinamide induced diabetic rats: quercetin as a potential antidiabetic agent acting via 11beta-hydroxysteroid dehydrogenase type 1 inhibition. *Eur J Med Chem.* 2010;45(6):2606–2612. 10.1016/j.ejmech.2010.02.049 20346546

[27] Kim JH Kang MJ Choi HN: Quercetin attenuates fasting and postprandial hyperglycemia in animal models of diabetes mellitus. *Nutr Res Pract.* 2011;5(2):107–111. 10.4162/nrp.2011.5.2.107 21556223 PMC3085798

[28] Zhang Y Qian Q Ge D: Identification of benzophenone *C*-glucosides from mango tree leaves and their inhibitory effect on triglyceride accumulation in 3T3-L1 adipocytes. *J Agric Food Chem.* 2011;59(21):11526–11533. 10.1021/jf2028494 21923172

[29] Andayani R Maimunah Yovita L: Penentuan antioksidan, kadar fenolat total dan likopen pada buah tomat ( *Solanum lycopersicum* L). *Jurnal Sains dan Teknologi Farmasi.* 2008;13(1):31–37. Reference Source

[30] Xu BJ Chang SK: A comparative on phenolic profiles and antioxidant activities of legumes as affected by extraction solvents. *J Food Sci..* 2007;72(2):S159–S166. 10.1111/j.1750-3841.2006.00260.x 17995858

[31] Musa KH Abdullah A Jusoh K: Antioxidant activity of pink-flesh guava ( *Psidium guajava L*.): effect of extraction techniques and solvents. *Journal of Food Analytical Methods.* 2011;4:100–107. 10.1007/s12161-010-9139-3

[32] Molyneux P: The use of the stable free radical diphenylpicrylhydroxyl (DPPH) for estimating antioxidant activity. *Songklanakarin Journal of Clinical Nutrition.* 2004;78(3):570S–578S. Reference Source

[33] Robinson RG Starr LB Kubos KL: A two-year longitudinal study poststroke mood disorder: findings during the initial evaluation. *Stroke.* 1983;14(5):736–741. 10.1161/01.str.14.5.736 6658957

[34] Richa Y: Radical Capture Activity Test of Extracts Petroleumeter, Ethyl Acetate and Ethanol Rhizoma Binahong (Anredera cordifolia (Tenore) Steen) with DPPH (2,2-diphenyl-1pikrihydrazil) Method).Faculty of Pharmacy, Muhammadiyah University, Surakarta,2009.

[35] Badarinath AV Mallikarjuna RAo K Madhu Sudhana Chetty C: A review on *In-Vitro* Antioxidant Methods: Comparison, Correlations and Consideration. *International Journal of Pharmaceutics Technology Research.* 2010;2(2):1276–1285. Reference Source

[36] De A Chattopadhyay S: The variation in cytoplasmic distribution of mouse peritoneal macrophage during phagocytosis modulated by mangiferin, an immunomodulator. *Immunobiology.* 2009;214(15):367–376. 10.1016/j.imbio.2008.10.001 19362682

[37] Islam MR Mannan MA Kabir IA: Analgesic, anti-inflammatory and antimicrobial effects of ethanol extracts of mango leaves. *Journal of the Bangladesh Agricultural University.* 2010;8(2):239–244. 10.3329/jbau.v8i2.7932

[38] Amrita B Liakot A Masfida A: Studies on the antidiabetic effects of *Mangifera indica* stem-barks and leaves on nondiabetic, type 1 and type 2 diabetic model rats. *Bangladesh Journal of Pharmacology.* 2009;4(2):110–114. 10.3329/bjp.v4i2.2488

[39] Fitmawati F Juliantari E Saputra A: The Potential of Wild Mango Leaves from Sumatera as the Immunostimulant Agents. *Biosaintifika: Journal of Biology and Biology Education.* 2018;10(3):671–677. 10.15294/biosaintifika.v10i3.16549

[40] Okwu D Ezenagu V: Evaluation of the phytochemical composition of mango ( *Mangifera Indica* Linn) stem bark and leaves. *International Journal of Chemical Science.* 2008;6(2):705–716. Reference Source

[41] Ou B Huang D Hampsch-Woodill M: When east meets west: the relationship between yin-yang and antioxidation-oxidation. *FASEB J.* 2003;17(2):127–129. 10.1096/fj.02-0527hyp 12554690

[42] Akowuah GA Ismail Z Norhayati I: Sinensetin, eupatorin, 3’hydroxy-5,6,7,4’-tetramethoxyflavone and rosmarinic acid contents and antioxidant effect of *Orthosipon stamineus* from Malaysia. *Food Chemistry.* 2004;87(4):559–566. 10.1016/j.foodchem.2004.01.008

[43] Ramírez NM Farias LM Santana FA: Extraction of Mangiferin and Chemical Characterization and Sensorial Analysis of Teas from *Mangifera indica* L. Leaves of the Ubá Variety. *Beverages.* 2016;2(4): 33, 1–13. 10.3390/beverages2040033

[44] Cornelia M Sutisna JA: Pemanfaatan daun mangga arum manis ( *Mangifera indica l*.) sebagai minuman teh celup. *FaST - Jurnal Sains Dan Teknologi.* 2019;3(1):71–81. Reference Source

[45] Ali MR Yong MJ Gyawali R: Mango ( *Mangifera indica* L.) peel extracts inhibit proliferation of HeLa human Cervical Carcinoma Cell via induction of Apoptosis. *Journal of Korean Social Applied Biological Chemistry.* 2012;55:397–405. 10.1007/s13765-012-1024-x

[46] Wauthoz N Balde A Balde ES: Ethnopharmacology of *Mangifera indica* L. bark and pharmacological studies of its main C-glucosylxanthone, Mangiferin. *International Journal of Biomedical and Pharmaceutical Sciences.* 2007;1(2):112–119. Reference Source

[47] Shivashankara K Isobe S Al-Haq M: Fruit antioxidant activity, ascorbic acid, total phenol, quercetin, and carotene of Irwin mango fruits stored at low temperature after high electric field pretreatment. *J Agric Food Chem.* 2004;52(5):1281–1286. 10.1021/jf030243l 14995134

[48] Soong YY Barlow PJ: Quantification of gallic acid and ellagic acid from longan ( *Dimocarpus longan* Lour.) seed and mango ( *Mangifera indica* L.) kernel and their effects on antioxidant activity. *J Food Chem.* 2006;97(3):524–530. 10.1016/j.foodchem.2005.05.033

[49] Núñez Sellés AJ Vélez Castro HT Agüero-Agüero J: Isolation and quantitative analysis of phenolic antioxidants, free sugars, and polyols from mango ( *Mangifera indica* L.) stem bark aqueous decoction used in Cuba as a nutritional supplement. *J Agric Food Chem.* 2002;50(4):762–6. 10.1021/jf011064b 11829642

[50] Ahmed A Saeid D Eman A: Egyptian mango by-product 1. Compositional quality of mango seed kernel. *J Food Chem.* 2007;103(4):1134–1140. 10.1016/j.foodchem.2006.10.017

[51] Schieber A Ullrich W Carle R: Characterization of polyphenols in mango puree concentrate by HPLC with diode array and mass spectrometric detection. *Innov Food Sci Emerg Technol.* 2000;1(2):161–166. 10.1016/S1466-8564(00)00015-1

[52] Velderrain-Rodríguez GR Torres-Moreno H Villegas-Ochoa MA: Gallic Acid Content and an Antioxidant Mechanism Are Responsible for the Antiproliferative Activity of ‘Ataulfo’ Mango Peel on LS180 Cells. *Molecules.* 2018;23(3): pii: E695. 10.3390/molecules23030695 29562699 PMC6017175

[53] Ribeiro S Schieber A: Bioactive compounds in mango ( *Mangifera indica* L.) *Bioactive Foods in Promoting Health.* 2010;34:507–523. 10.1016/B978-0-12-374628-3.00034-7

[54] Madsen HL Bertelsen G: Spices as antioxidants. *Trends in Food Science and Technology.* 1995;6(8):271–277. 10.1016/S0924-2244(00)89112-8

[55] Kim SH Jun CD Suk K: Gallic acid inhibits histamine release and pro-inflammatory cytokine production in mast cells. *Toxicol Sci.* 2006;91(1):123–131. 10.1093/toxsci/kfj063 16322071

[56] Ajila CM Prasada RU: Mango peel dietary fibre: Composition and associated bound phenolics. *Journal of Fungtional Food.* 2013;5(1):444–450. 10.1016/j.jff.2012.11.017

[57] Singh D Chander V Chopra K: Quercetin, a bioflavonoid, attenuates ferric nitrilotriacetate-induced oxidative renal injury in rats. *Drug Chem Toxicol.* 2004;27(2):145–156. 10.1081/dct-120030729 15198074

[58] Loke WM Hodgson JM Proudfoot JM: Pure dietary flavonoids quercetin and (-)-epicatechin augment nitric oxide products and reduce endothelin-1 acutely in healthy men. *Am J Clin Nutr.* 2008;88(4):1018–1025. 10.1093/ajcn/88.4.1018 18842789

[59] Zahedi M Ghiasvand R Feizi A: Does Quercetin Improve Cardiovascular Risk factors and Inflammatory Biomarkers in Women with Type 2 Diabetes: A Double-blind Randomized Controlled Clinical Trial. *Int J Prev Med.* 2013;4(7):777–785. 24049596 PMC3775217

[60] Lamson DW Brignall MS: Antioxidants and cancer. III: Quercetin. *Altern Med Rev.* 2000;5(3):196–208. 10869101

[61] Molina MF Sanchez-Reus I Iglesias I: Quercetin, a flavonoid antioxidant, prevents and protects against ethanol-induced oxidative stress in mouse liver. *Biol Pharm Bull.* 2003;26(10): 1398–1402. 10.1248/bpb.26.1398 14519943

[62] Fitmawati Juliantari E Roza RM: Antioxidant Activity of Wild Mango (Mangifera) from Sumatra. Proceeding Applied Science and Technology. *Dissemination in International Conference Science and Technology.*Oct; 292018; Pekanbaru, Indonesia.

[63] Syahputra RF Resida E Kholifah SN: Wild Mango Exploration in Sumatra for Potential Health Medicine.2020 10.17605/OSF.IO/MP6KF

