# Supplementary material for: Phytochemical screening and antioxidant profiling of Sumatran wild mangoes ( Mangifera spp.): a potential source for medicine antidegenerative effects
Source: F1000Res. 2020 Jun 30;9:220. Originally published 2020 Mar 31. [Version 3] doi: 10.12688/f1000research.22380.3 (PMC7309414; doi:10.12688/f1000research.22380.3)
Supplement: Peer review history

# Peer review: Reviewer response for version 2

**DOI:** 10.5256/f1000research.26611.r64334

**Published:** 2020-06-22

**Authors:** Kamal Rullah

**Affiliations:** 1 Kulliyyah of Pharmacy, International Islamic University Malaysia, Kuantan, Malaysia

**Recommendation**

 The manuscript by Fitmawati and co-workers describes the antioxidant profiling of Sumatran wild mangoes ( *Mangifera spp.*). I noted that the manuscript had been revised from the previous submission. Overall, it is a more organized and constructed paper that reports the findings from a sound and interesting study. This manuscript showed a good similarity checker as well. Thus, the manuscript may be recommended for publication in F1000Research after some revision.

1. The author(s) need to revise the title of the manuscript. The term ‘antidegenerative medicine’ is uncommon in a scientific paper. There are no antidegenerative medicine and antidegenerative drug as well, but there is a term ‘antidegenerative effect’. Hence, I suggest that the title should be ‘…..Phytochemical Screening and Antioxidant Profiling of Sumatran Wild Mangifera Species…..”.
2. Why the author(s) decided that the compound gallic acid and quercetin are representing the phenolic and flavonoid compound, respectively? Based on the reference Barreto and co-worker ( https://doi.org/10.1021/jf800738r and the author(s) have cited the paper), the major phenolic compound was mangiferin, a precise compound isolated from Mangifera. In my view, the mangiferin is a more noteworthy compound from Mangifera species. Moreover, there is no report on quercetin as the aglycone form (as the author(s) applied as a standard compound) in Mangifera species. Thus, I suggest the author(s) verify the qualitative component of both ethanolic extract, leaves, and bark, by using the Liquid chromatography-Mass Spectrometry (LC-MS). Check whether the standard compound exists in the desired ethanolic extract by mass spectrometry. This is necessary to ensure the selection of the specific standard compound.
3. The statistical data is missing. The author(s) should provide extensive statistical analysis in this manuscript, i.e. standard deviation (SD) is missing in Table 1, Table 2, and Table 3 as well. While presenting research data, the author(s) should be aware of using adequate statistical measures. 
4. The validation of the analytical procedures of HPLC is missing, including accuracy, precision, specificity, etc.
5. The author(s) needs to improve the conclusion. The manuscript was not employed Mangifera species as an immunomodulatory agent.

Is the work clearly and accurately presented and does it cite the current literature?

Yes

If applicable, is the statistical analysis and its interpretation appropriate?

Partly

Are all the source data underlying the results available to ensure full reproducibility?

Yes

Is the study design appropriate and is the work technically sound?

Yes

Are the conclusions drawn adequately supported by the results?

Partly

Are sufficient details of methods and analysis provided to allow replication by others?

Yes

Reviewer Expertise:

Medicinal Chemistry and Drug Discovery

I confirm that I have read this submission and believe that I have an appropriate level of expertise to confirm that it is of an acceptable scientific standard.

# Peer review: Reviewer response for version 1

**DOI:** 10.5256/f1000research.24692.r61846

**Published:** 2020-05-04

**Authors:** Sarifah Nurjanah

**Affiliations:** 1 Department of Agriculture Engineering and Biosystem, Faculty of Agro-Industrial Technology, Padjadjaran University , Bandung, Indonesia

The authors describe in detail a new information on antioxidant profile of 11 Sumatran wild mangoes ( *Mangifera *spp), including flavonoid and phenolic content as well as gallic acid and quercetin of its leaves and barks. The paper is well written. A few suggestions are made, however:

**Introduction**: Need information why antioxidant activity is determined on leaves and barks instead of mango’s peel, pulp and seed kernel.

**Method **:

1. There is no information about quercetin and gallic acid standard for HPLC analysis. Need to specify the origin of quercetin and gallic acid standards.
2. No information of HPLC column, please specify the column used.

**Results and discussion** :

1. The last sentence of paragraph 6 stated “Ailla and Prasada ^46^ stated that quercetin is the major flavonoid type in the peel of mango”, since this paragraph talk about gallic acid not quercetin, so discussion about quercetin should be moved in the paragraph 8.
2. On paragraph 8 stated the lowest quercetin was found in *M foetida* (var Limus), actually as we can see from Table 3, it should be *M foetida _2_* (var. manis) and *M foetida _3_* (var. batu).
3. Total phenolic is represented by gallic acid content. Increasing phenolic content should increase the gallic acid, but the study didn’t show this. Why this condition can appear? Does any others phenolic compound instead of gallic acid influence the total phenolic in mango barks and leaves?
4. Flavonoid content is represented by quercetin. Increasing flavonoid content should increase the quercetin, but the study didn’t show this. Why this condition can appear? Does any others flavonoid compound instead of quercetin influence the flavonoid compound in mango barks and leaves?
5. IC _50_ values indicate the antioxidant activity, the lower IC _50_ value the higher antioxidant activity. In this study the antioxidant activity is represented by total phenolic and flavonoid content. Lowering IC _50_ should increase total phenolic and flavonoid content, but the study didn’t show this. Please discuss this phenomena.

Is the work clearly and accurately presented and does it cite the current literature?

Yes

If applicable, is the statistical analysis and its interpretation appropriate?

Yes

Are all the source data underlying the results available to ensure full reproducibility?

Yes

Is the study design appropriate and is the work technically sound?

Yes

Are the conclusions drawn adequately supported by the results?

Yes

Are sufficient details of methods and analysis provided to allow replication by others?

Yes

Reviewer Expertise:

Active compound of agriculture product

I confirm that I have read this submission and believe that I have an appropriate level of expertise to confirm that it is of an acceptable scientific standard.

# Response

**Published:** 2020-05-13

**Authors:** Fitmawati Fitmawati

**Affiliations:** Universitas Riau, Indonesia

We would to thank the reviewer for their comments to our manuscript. In this new version, we have reviewed and modified text to improve the clarity and understanding manuscript. The main changes have been:

1). Introduction the reason for using leaves and bark to determine the antioxidant value of wild mangoes is added.

2). The method has already mentioned the origin of the standard quercetin and gallic acid and the HPLC column used.

3). In the results and discussion of data changes due to input errors in the previous article. It has been discussed that there are other compounds of flavonoid and phenolic derivatives in leaves and bark of wild mango that refer to the literature, thus affecting the amount of gallic acid and quercetin, as well as antioxidant values.
